# Microbial Flora Associated with the Halophyte–*Salsola imbricate* and Its Biotechnical Potential

**DOI:** 10.3389/fmicb.2018.00065

**Published:** 2018-01-31

**Authors:** Fehmida Bibi, Gary A. Strobel, Muhammad I. Naseer, Muhammad Yasir, Ahmed A. Khalaf Al-Ghamdi, Esam I. Azhar

**Affiliations:** ^1^Special Infectious Agents Unit, King Fahd Medical Research Center, King Abdulaziz University, Jeddah, Saudi Arabia; ^2^Department of plant sciences, Montana State University, Bozeman, MT, United States; ^3^Center of Excellence in Genomic Medicine Research, King Abdulaziz University, Jeddah, Saudi Arabia; ^4^Department of Medical Laboratory Technology, Faculty of Applied Medical Sciences, King Abdulaziz University, Jeddah, Saudi Arabia

**Keywords:** halophyte, antifungal activity, enzyme production, 16S rRNA gene sequence, LC/MS analyses

## Abstract

Halophytes are associated with the intertidal forest ecosystem of Saudi Arabia and seemingly have an immense potential for yielding useful and important natural products. In this study we have aimed to isolate and characterize the endophytic and rhizospheric bacterial communities from the halophyte, *Salsola imbricata*, In addition these bacterial strains were identified and selected strains were further studied for bioactive secondary metabolites. At least 168 rhizspheric and endophytic bacteria were isolated and of these 22 were active antagonists against the oomycetous fungal plant pathogens, *Phytophthora capsici* and *Pythium ultimum*. Active cultures were mainly identified with molecular techniques (16S r DNA) and this revealed 95.7–100% sequence similarities with relevant type strains. These microorgansims were grouped into four major classes: *Actinobacteria, Firmicutes*, β*-Proteobacteria*, and γ*-Proteobacteria*. Production of fungal cell wall lytic enzymes was detected mostly in members of *Actinobacteria* and *Firmicutes*. PCR screening for type I polyketide synthases (PKS-I), type II polyketide synthases (PKS-II) and nonribosomal peptide synthetases (NRPS) revealed 13 of the 22 strains (59%) were positive for at least one of these important biosynthetic genes that are known to be involved in the synthesis of important antibiotics. Four bacterial strains of *Actinobacteria* with potential antagonistic activity including two rhizobacteria, EA52 (*Nocardiopsis* sp.), EA58 (*Pseudonocardia* sp.) and *two* endophytic bacteria *Streptomyces sp*. (EA65) and *Streptomyces* sp. (EA67) were selected for secondary metabolite analyses using LC-MS. As a result, the presence of different bioactive compounds in the culture extracts was detected some of which are already reported for their diverse biological activities including antibiotics such as Sulfamethoxypyridazine, Sulfamerazine, and Dimetridazole. In conclusion, this study provides an insight into antagonistic bacterial population especially the *Actinobacteria* from *S. imbricata*, producing antifungal metabolites of medical significance and characterized taxonomically in future.

## Introduction

Coastal dune plants consist of shrubs that are associated with tropical and subtropical coastal areas. This ecosystem is considered as a low nutrient environment due to the sparseness of nitrogen and phosphorus (Frosini et al., 2012). Coastal dune areas of the world usually represent a unique flora as a result of variations in salinity, anaerobicity, and temperature fluctuations during different seasons. All of these conditions make this habitat unusual and thus may have been selective for the presence of truly unique microorganisms having a rich biodiversity. Under these conditions microorganisms associated with these costal dune plants play a role in their survival and perform diverse biological functions in plants (Del Vecchio et al., 2015).

Bacteria associated with Coastal dune plants are responsible for various activities including resistance against different pathogens by producing different bioactive metabolites and plant growth promotion compounds. Bacteria are associated with these plants either as rhizospheric or endophytic forms and use nutrients from plants for their growth (de Melo Pereira et al., 2012). Endophytic bacteria get access to internal parts of plants by producing enzymes to hydrolyze cell wall of plants (Cho et al., 2007). These hydrolytic enzymes are also involved in defense of plants against various bacterial and fungal pathogens (Luo et al., 2013).

Endophytes produce metabolites that possess antimicrobial activities (Strobel, 2003). Due to their beneficial effects and broad range, endophytes have been isolated from plants widely ranging from the poles of the earth to the steaming tropics. Plants provide these microorganisms, particularly endophytic bacteria and fungi, a nutrient-rich environment for their growth and development (Strobel et al., 2004; de Melo Pereira et al., 2012). Endophytic *Actinobacteria* associated with plant roots or leaves are a source of novel potential bioactive substances. *Actinobacteria* produce various secondary metabolites including antibiotics, antitumor, immunosuppressive, and enzymes widely used in industrial, agriculture, and pharmaceutical industry (Manivasagan et al., 2014). Recently, various antimicrobial compounds and enzymes have been isolated from marine *Actinobacteria* (Hong et al., 2009; He et al., 2012). Secondary metabolite salinosporamide A, isolated from a marine *Actinobacteria* is currently in clinical trials as a cancer treatment (Feling et al., 2003). Several other bioactive compounds have been isolated from different marine *Actinobacteria* (Fu et al., 2012).

The Genus *Salsola* is comprised of different halophyte species including *Salsola imbricata* that inhabit the coastal area of the Thuwal region in Jeddah, Saudi Arabia. Recent studies on *S. imbricata* reported the presence of triterpenesaponins and a new isorhamnetin from the methanolic extract of this plant (Osman et al., 2016). In a recent study from Saudi Arabia, phytochemical analysis of *S. imbricata* showed presence of bioactive biphenylpropanoids i.e., biphenylsalsonoid A and B. These two bioactive compounds exhibited antioxidant and antibacterial activity against human pathogenic bacteria (Oueslati et al., 2017). Although the coastal dune environment is an unexplored source of natural compounds with diverse activities there has been little or no work on the bacterial flora of this plant. Based on previous studies, it would appear that certain microbial flora components may be present in this plant that has biotechnical potential. Thus, this study was undertaken to explore potential antifungal microflora of halophyte that may produce products of medicinal, agricultural, and industrial significance.

## Materials and methods

### Collection of plant samples and isolation of rhizobacteria and endophytic bacteria

The halophyte, *S. imbricata* was collected from the coastal area of Jeddah, Saudi Arabia (22° 15′ 54″ North, 39° 6′ 44″ East). We used soil, roots and leaves of plant sample for the isolation of both rhizopheric and endophytic bacteria and the sample plant were placed in sterile plastic bag at the time of collection. For isolation of bacteria from soil associated with roots they were dipped in sterile distilled water and serial dilutions were made (10^−3^, 10^−4^, and 10^−5^) in filtered autoclaved sea water (FAS) and spread in triplicate on four different media used for culturing of bacteria. We have used half strength R2A (½ R2A) [0.25 g yeast extract, 0.25 g proteose peptone No. 3 (Difco), 0.25 g casamino acid, 0.25 g dextrose, 0.25 g soluble starch, 0.15 g sodium pyruvate, 0.15 g K_2_HPO_4_, 0.03 g MgSO_4_], half Tryptic soy agar (½ TSA) [Pancreatic Digest of Casein, 7.5g Papain Digest of Soybean, 2.5 g Sodium Chloride, 2.5 g Agar, 15.0 g], marine agar (MA) [Peptone, 5.0 g Yeast Extract, 1.0 g Ferric Citrate, 0.1 g Sodium Chloride, 19.45 g Magnesium Chloride, 8.8 g Sodium Sulfate, 3.24 g Calcium Chloride, 1.8 g Potassium Chloride, 0.55 g Sodium Bicarbonate, 0.16 g Potassium Bromide, 0.08 g Strontium Chloride, 34.0 mg Boric Acid, 22.0 mg Sodium Silicate, 4.0 mg Sodium Fluoride, 2.4 mg Ammonium Nitrate, 1.6 mg Disodium Phosphate, 8.0 mg Agar,15.0 g] and half nutrient agar (½ NA) [Beef Extract, 1.5 g Peptone, 2.5 g Agar, 15.0 g] (Difco Laboratories, Detroit, MI) in 1000 ml filtered Sea water for bacterial culturing. For isolation of endophytic bacteria, roots, and leaves were surface sterilized. Both roots and leaves were washed several times with tap water to remove adhering soil particles and then sterilized by washing with disinfectants as described previously (Bibi et al., 2012). After sterilization, small pieces of sterilized root and leaf segments were ground in FAS using a sterile mortar with pestle. Aliquots were further serially diluted and plated in triplicate on four different media mentioned above. To suppress fungal growth, 50 μg/ml cycloheximide was mixed to the medium before pouring. The plates were incubated at 28°C for 2 weeks for bacterial growth.

### Isolation of rhizopheric and endophytic Actinobacteria

For the isolation of rhizosphere and endophytic *Actinobacteria*, the same dilutions from soil, roots, and leaves were inoculated onto starch-casein agar (Himedia) in sea water supplemented with cycloheximide and nystatin (50 μg/ml). Further, plates were incubated at 28°C for up to 10-12 days. By checking morphological characteristics, *Actinobacteria* were selected and subcultured using the same isolation medium. Individual colonies were streaked to check purity of the strains and stored in 15% (v/v) glycerol. All bacterial strains were labeled according to acquisition numbers of the King Abdulaziz University special infectious agents unit and stored at −70 C.

### Screening of bacteria for antifungal activity

All isolated bacteria were screened for their antifungal activity. We used five different fungal pathogens i.e., *Phytophthora capsici* (*P. capsici*), *Pythium ultimum (Py. ultimum)*, (obtained in this laboratory from *Capsicum annuum* and *Cucumis sativus* respectively)*, Magnaporthe grisea* (KACC 40415), *Altenaria malli* (KCTC 6972) and *Fusarium moniliforme* (KCTC 6149) were obtained from Korean Agricultural Culture Collection (KACC) and Korean type culture collection center (KCTC). Antifungal activity was determined by using the cross streak method (Bibi et al., 2012). All isolates were streaked on PDA medium supplemented with ½ R2A in sea water. Each 6 mm mycelial disc of freshly grown fungal pathogen was placed in the center of plate perpendicular to streak of isolates at 4 cm distance from edges of plate and incubated for 4–6 days at 28°C. Antagonistic strains were checked in duplicate for antagonistic activity evaluated by measuring the inhibition zone of fungal pathogens around each bacterial streak.

### Bacterial DNA extraction and 16S rRNA gene sequencing

Genomic DNA was extracted from the selected antagonistic bacterial isolates using a DNA extraction kit (Thermo Scientific, Waltham, USA). To identify antagonistic bacteria, 16S rRNA gene sequencing was performed. Approximately 1500 bp fragment of the 16S rRNA gene was amplified using bacterial universal primers 27F (5′-AGAGTTTGATCCTGGCTCAG-3′) and 1492R (5′- GGTTACCTTGTTACGACTT-3′), the 16S rRNA gene fragment was amplified under the following PCR conditions: one cycle of 95°C for 5 min followed by 28 cycles of 95°C for 1 min, and annealing at 58°C for 50 s with extension at 72°C for 50s, and a final extension step at 72°C for 10 min (Bibi et al., 2012). PCR products were purified using PCR purification kit (Thermo Scientific, Waltham, USA), and sequenced commercially (Macrogen, South Korea). Antagonistic bacteria were identified by blast searches of their 16S rRNA gene sequences obtained using the EzTaxon server (https://www.ezbiocloud.net) (Kim et al., 2012) to identify antagonistic bacteria. Phylogenetic positions of the antagonistic bacteria were confirmed using CLUSTALX (Thompson et al., 1997) multiple alignments of the bacterial sequences and BioEdit software (Hall, 1999) was used to edit the gaps. The neighbor-joining method in the MEGA6 Programme with bootstrap values based on 1,000 replications was used for construction of the phylogenetic tree based on the 16S rRNA gene sequences (Tamura et al., 2013).

### Evaluation of hydrolytic enzymatic activity

Antagonistic bacterial isolates were further evaluated for their lytic enzyme production. Protease activity was checked using skim milk ½ R2A agar plates. Bacteria exhibiting protease production made clear zone on skim milk agar plates. Amylase production was checked on starch medium. Starch hydrolysis was seen as a clear zone on agar plates by positive strains (Kumar et al., 2012). For lipase activity, tributyrin ½ R2A agar medium was used. After 48 h of incubation at 28°C a clear zone was detected around bacterial colonies after hydrolysis of tributyrin. To check cellulase activity, CMC agar (carboxy methyl cellulose agar) medium was used. Bacteria were streaked on plates with the respective enzyme medium and incubated at 28°C for 2 days. Congo red (0.1%) solution was added to plates and put on orbital shaker for 30 min and then rinsed with 1MNaCl (Hendricks et al., 1995). Cellulase activity was observed as a clear zone on CMC agar plates around bacterial colonies.

### Detection of antimicrobial genes

It has been well established that many antimicrobial products contain polyketides. Thus, to determine if the antagonistic bacteria might contain such enzymes, a search of the bacterial genome was done to check for the presence of genes coding for these enzymes. To detect biosynthetic genes, polyketide synthetase I (PKS-I) and nonribosomal peptide synthetase (NRPS) genes, the following primer pairs K1F/M6R (5′-TSAAGTCSAACATCGGBCA-3′; 5′-CGCAGGTTSCSGTACCAGTA-3′) and A3F/A7R (5′GCSTACSYSATSTACACSTCSGG-3′; 5′-SASGTCVCCSGTSCGGTAS-3′), respectively were used (Ayuso-Sacido and Genilloud, 2005). Amplification of polyketide synthetase II (PKS-II) gene was performed using primer pair KSα/Kβ (5′-TSGCSTGCTTGGAYGCSATC-3′; 5′-TGGAANCCGCCGAABCCTCT-3′) (Metsa-Ketela et al., 1999). PCR reaction mixture (25 μl) contained 12.5 μl of PCR mastermix (promega), 1 μl of each primer, 1 μl of template DNA, and 9.5 μl of Nuclease-Free Water. Amplifications were performed under following PCR conditions: one cycle of 95°C for 5 min followed by 28 cycles of 95°C for 1 min, and annealing at 58°C (K1F/M6R), 56°C (A3F/A7R) and 62°C (KSα/Kβ) for 50 s with extension at 72°C for 50 s, and a final extension step at 72°C for 10 min. PCR amplification products were then checked by using 0.8% agarose through gel electrophoresis, and bands of 1.2–1.4 kb, 600 bp, and 700–800 bp were detected as products of PKS-I, PKS-II, and NRPS genes, respectively.

### Optimization of bacterial culture conditions for production bioactive compounds

To optimize culture conditions of selected bacterial strains four different media i.e., ½ R2A broth, ½ TSB, ½NB in sea water and Marine broth in distilled were used for culturing. Bacterial cultures were grown for 72 h and after every 24 h optical density (OD) was checked and antifungal activity was assessed against *P. capsici, Py. ultimum* using the disc diffusion method. The effect of temperature was optimized within the range of temperatures (20–40°C) in ½ R2A broth. For pH optimization, different ranges of pH values (5–12) were used for the growth and antifungal compound production in ½ R2A broth.

### LC-MS analysis of bacterial culture

In order to assess the actual presence of antimicrobial products in bacterial cultures that had been previously screened and shown to be biologically active we performed LC-MS analyses of the bacterial supernatants. A Bacterial culture (5 ml) was placed on −80°C for 5 min, and then transfer to 37°C water bath for 5 min and this procedure was repeated 5 times. It was centrifuged at 15,000 g for 10 min and transfer 3 ml supernatant fluid was transferred to a tube. To it was added 12 ml of acetonitrile and vortexed for 30 s. It was centrifuged again at 15,000 g for 10 min and 300 μl supernatant was taken for LC-MS metabolomic analysis. The injection volume was 3 μl and samples were analyzed on an Agilent 6540B TOF/Q-TOF Mass Spectrometer coupled with an Agilent 1290 UPLC and Dual AJS ESI ion source. An ACQUITY UPLC HSS T3 (100 × 2.1mm, 1.8 um) column and pre-column (Phenomenex Security Guard™) was used to separate the sample. The column temperature was set to 45°C and flow rate was 0.5 ml/min. The acquisition range was from 50 m/z to 1500 m/z and the scan rate was 1.00 spec/sec. MS conditions were set as follow: capillary voltage 3500 V, nebulizer pressure 35 psi, drying gas 10 L/min, gas temperature 325°C, vaporizer 200 V, voltage charge 1000 V; negative-ion mode capillary voltage 3500 V, corona negative 15.0 V, fragmentor 175 V, skimmer1 65.0 V, octopole RF Peak 750 V; positive ion mode capillary voltage 3500 V, corona positive 4.0 V, fragmentor 175 V, skimmer1 65.0 V, and octopole RF Peak 750 V. Raw data was imported to the Agilent MassHunter Qualitative Analysis B.06.00 software. Metabolites were identified by using National Institute of Standards and Technology database (NIST) database. Further bioactive metabolites from other secondary metabolites in culture extract were identified using different public databases such as PubChem (http://pubchem.ncbi.nlm.nih.gov/), ChemSpider (http://www.chemspider.com/), SciFinder (http://www.cas.org/products/scifinder), and ChEMBL (https://www.ebi.ac.uk/chembl/).

### Nucleotide sequence numbers

Nucleotide sequences of the antagonistic bacteria isolated from the halophyte have been deposited in the GenBank database under accession numbers KY436424–KY436445.

## Results

### Isolation of rhizospheric and endophytic bacteria

In this study, rhizospheric and endophytic bacteria were isolated from the halophyte, *S. imbricata* growing in a selected site on the Saudi Arabian peninsula. A total of 168 rhizospheric and endophytic bacteria were isolated from soil, roots, and leaves samples. Various methods were used to acquire these bacteria including the use of four different culturing media. It turns out that both high and low nutrient medium content favored the growth of different groups of bacteria. It has been noted that the ½ R2A in SW is a favorable medium for the isolation of bacteria from the marine environment. Thus, all isolated bacteria culture on different media were sub-cultured on ½ R2A in SW. In total 168 bacteria were isolated from different parts of halophyte and soil as well (Table 1). From soil, 85 rhizobacteria were isolated by culturing on different media listed above for isolation. Roots samples used for isolation of endophytic bacteria yielded 48 endophytic bacteria. While from leaves 35 bacteria were recovered. Details of bacteria number their antagonistic activity and dominance of different bacterial phyla is mentioned in Table 1.

**Table 1:** Distribution of rhizopheric and endophytic antagonistic bacteria isolated from halophyte.

**Isolation source^a^**	**Number of isolates^b^**	**Number of antagonist^c^**	**Antagonists (%)^d^**	**Dominant phylum^e^**
Rhizopheric (Soil)	85	8	4.7	*Actinobacteria*
Endophytes (Roots)	48	9	5.4	*Actinobacteria*
Endophytes (Leaves)	35	5	2.9	*Firmicutes*
	168	22	13%	*Actinobacteria*

a *Isolation source of rhizopheric and endophytic bacteria*.

b *Total number of rhizopheric and endophytic bacteria isolated from soil, roots and leaves of halophyte*.

c *Total number of antagonistic rhizopheric and endophytic bacteria*.

d *Percentage of antagonistic rhizopheric and endophytic bacteria*.

e *Dominant phylum in all rhizopheric and endophytic antagonistic bacteria*.

### Screening of bacteria against pathogenic fungi

All isolated bacteria were tested for their antagonism against plant pathogenic oomycetes. Of the 168 bacteria isolated, 22 (13%) displayed inhibition to both oomycete plant pathogens that was the established bioassay test. The percentage of antagonistic bacteria varied in different parts of halophyte, being highest in roots (*n* = 9; 5.3%), followed by soil (*n* = 8; 4.7%), and leaves (*n* = 5; 2.9%). These antagonistic bacterial isolates were tested further against three other fungal pathogens. Different groups of antagonistic bacteria were identified from *S. imbricata*, and *Actinobacteria* was the dominant phylum (Table 1. Both rhizospheric and endophytic antagonistic *Actinobacteria* i.e., *Nocardiopsis dassonvillei* (EA52), *Pseudonocardia* sp. (EA58), *Arthrobacter* sp. (EA59), and *Streptomyces* sp. (EA64) showed strong inhibition against the oomycetes (Table 2). Some of the antagonistic isolates (EA56, EA62, EA68-EA70) showed inhibition against oomycetes but did not show inhibition against other pathogenic fungi used (Table 2).

**Table 2:** Taxonomic identification, antifungal acitivity, and enzymes production of rhizo and endophytic bacteria from halophyte.

		**Antifungal activity against^a^**							**Enzymatic activities^b^**				**Detection of**		
**Lab no**	**Closely related type strain**	**Accession number**	**% identity**	***P. ultimum***	***P. capsici***	***M. grisea***	***A. mali***	***F. moniliforme***	**Protease**	**Amylase**	**Lipase**	**Cellulase**	**NRPS**	**PKS-I**	**PKS-II**
***Salsola imbricate***															
**RHIZOPHERIC (SOIL)**															
EA51	*Nocardioides luteus* KCTC 9575^T^	KY436424	99.8	W	W	−	−	−	++++	−	++++	−	+	−	−
EA52	*Nocardiopsis dassonvillei subsp. dassonvillei* DSM 43111^T^	KY436425	99.7	++++	+++	+	++	−	++++	−	−	+++	+	−	+
EA53	*Bordetella avium* ATCC 35086 ^T^	KY436426	98.9	++++	++++	+++	−	−	−	−	−	−	−	−	−
EA54	*Pseudomonas boreopolis* ATCC 33662^T^	KY436427	99.8	+	+	+	−	−	−	−	−	−	−	+	−
EA55	*Nocardioides luteus* KCTC 9575^T^	KY436428	100	+	W	−	+	−	−	−	++++	−	−	+	−
EA56	*Nocardioides deserti* SC8A-24 ^T^	KY436429	98.9	+	+	−	−	−	−	−	−	−	−	−	−
EA57	*Arthrobacter subterraneus* CH7 ^T^	KY436430	99.3	+	W	−	+	−	++++	++++	−	−	+	−	−
EA58	*Pseudonocardia carboxydivorans* Y8^T^	KY436431	100	++++	++++	+++	++++	+	−	−	−	−	−	+	−
**ENDOPHYTES (ROOTS)**															
EA59	*Arthrobacter subterraneus* CH7 ^T^	KY436432	99.9	++++	+++	+	−	−	++++	+++++	++++	−	−	−	−
EA60	*Bacillus licheniformis* ATCC 14580^T^	KY436433	99.4	+++	++	+	+++	++	++++	+++++	−	−	−	−	−
EA61	*Streptomyces enissocaesilis* NBRC 100763^T^	KY436434	99.4	+++	+	+	+++	−	+++	+++++	−	−	+	−	+
EA62	*Streptomyces phaeopurpureus* NRRL B-2260^T^	KY436435	98.8	+	W	−	−	−	−	−	−	−	−	−	−
EA63	*Streptomyces geysiriensis* NBRC 15413^T^	KY436436	100	+	++	−	−	−	+++	W	−	−	+	−	+
EA64	*Streptomyces violaceochromogenes* NBRC 13100 ^T^	KY436437	99.3	+++	+++	+++	−	−	−	−	++++	−	+	−	+
EA65	*Streptomyces diastatochromogenes* ATCC 12309^T^	KY436438	97.3	++	++	+	−	+	−	−	−	−	−	−	+
EA66	*Bacillus sonorensis* NBRC 101234^T^	KY436439	95.7	++	+	+	+++	+++	−	−	++++	−	+	−	−
EA67	*Streptomyces europaeiscabiei* KACC 20186^T^	KY436440	98.2	++	+	++	+++	+	+++	−	−	−	−	−	+
**ENDOPHYTES (LEAVES)**															
EA68	*Bacillus tequilensis* KCTC 13622^T^	KY436441	100	+	W	−	−	−	−	−	−	−	−	−	−
EA69	*Nocardioides luteus* KCTC 9575^T^	KY436442	99.1	+	+	−	−	−	−	+++++	++++	−	−	−	−
EA70	*Agromyces indicus* NIO-1018 ^T^	KY436443	100	++	+	−	−	−	−	++	−	−	−	−	−
EA71	*Bacillus halosaccharovorans* E33^T^	KY436444	98.9	+++	+	+	−	+++	−	−	−	−	−	−	−
EA72	*Bacillus subtilis subsp. inaquosorum* KCTC 13429^T^	KY436445	99.3	+	++	++	+	−	+++	−	+++	−	+	+	+

a *Antagonistic activity of all bacteria isolated in this study. The activity was measured after 3–5 days incubation at 28°C by measuring the clear zone of mycelial growth inhibition: W, weak; –, Negative; +, 3 mm; ++, between 4 and 6 mm; +++, between 7 and 9 mm; ++++, between 10 and 12 mm*.

b *Production of protease, amylase, lipase, and cellulase was determined by plate assay. Enzymatic activity was estimated as zone of halo formed around bacterial colonies: W, weak; −, Negative; +, 3 mm; ++, between 4 and 6 mm; +++, between 7 and 9 mm; ++++, between 10 and 12*.

### Identification and phylogenetic analysis of rhizopheric and endophytic antagonistic bacteria

**Figure 1:**
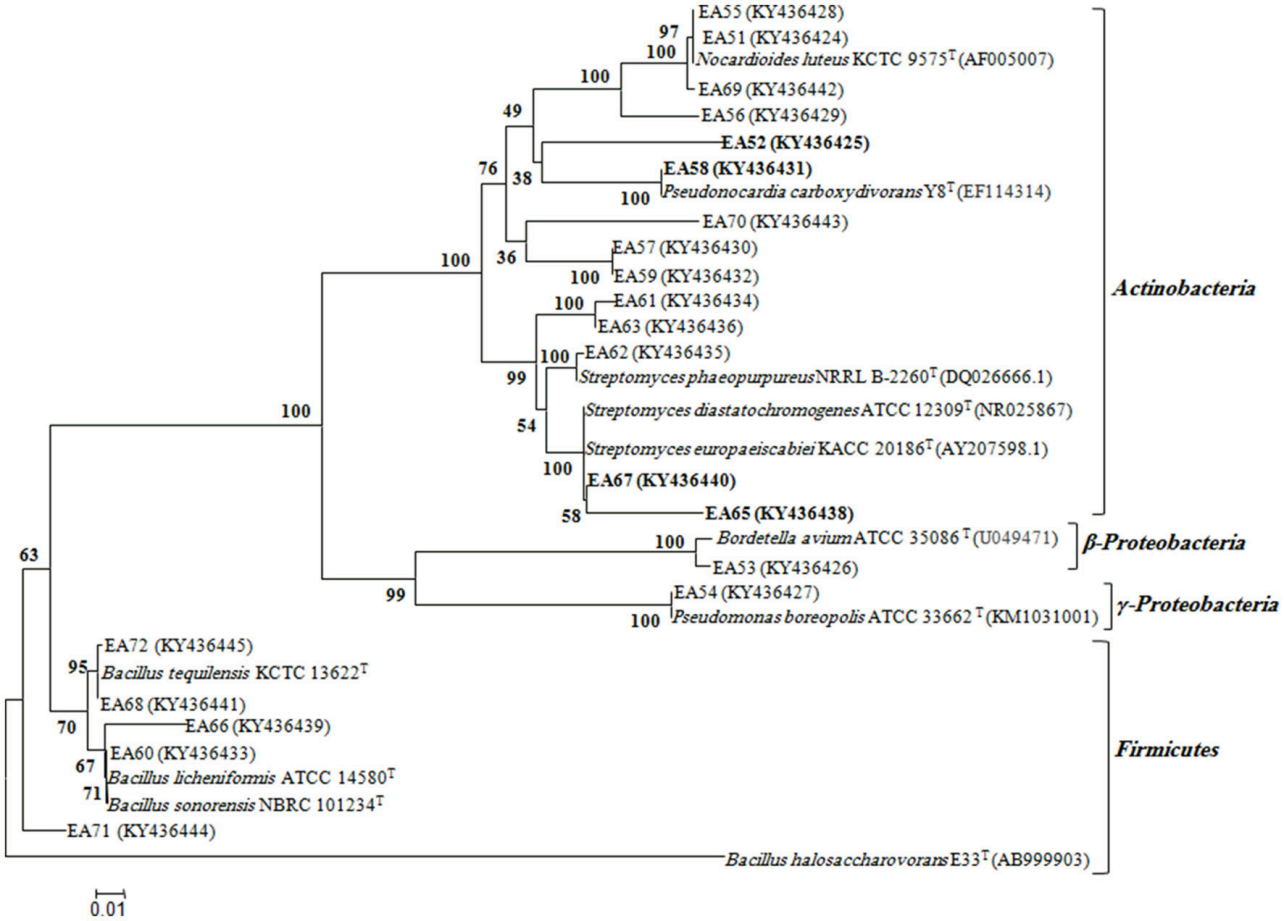
Phylogenetic distribution of antagonistic bacteria isolated from *S.imbricata* on the basis of 16S rRNA gene sequences obtained from bacteria and closely related sequences of the type strains of other species. The phylogenetic relationships were inferred from the 16S rRNA gene sequences (1 kb) by using the neighbor-joining method from distances computed with the Jukes-Cantor algorithm. Bootstrap values (1,000 replicates) are shown next to the branches. GenBank accession numbers for each sequence are shown in parentheses. Bar, 0.01 accumulated changes per nucleotide. Isolates selected for bioactive metabolites identification are highlighted.

In total, 22 antagonistic bacteria were identified by using 16S rRNA gene sequence analysis. Result of sequencing showed that eight different genera of bacteria were identified belonging to four major classes: *Actinobacteria* (*n* = 15; 68%), *Firmicutes* (*n* = 5; 23%), γ*-Proteobacteria* (*n* = 1; 4.5%), and β*-Proteobacteria* (*n* = 1; 4.5%). Sequence identities of antagonistic bacteria were recorded from 95.7−100% (Table 1). Sequencing results indicated *Actinobacteria* as the dominant phylum and 26% of the antagonistic isolates belonged to the genus *Streptomyces* (Table 1). Using 16S rRNA gene data, a neighbor Joining (NJ) phylogenetic tree was constructed (Figure 1). High bootstrap values were recovered in phylogenetic tree. A separate cluster has been generated for isolates of class *Actinobacteria*. Antagonistic strains in this cluster were identified with high bootstraps values (51–100%). Representatives of this class belong to four different genera i.e., *Nocardioides, Nocardiopsis, Arthrobacter*, and *Streptomyces*. One strain of β*-Proteobacteria* was identified with separate cluster also showing high bootstrap value (100%). One strain of γ*-Proteobacteria* made a distinct cluster with closely related type strain *Pseudomonas* sp. (EA54) with high a bootstrap value of 100 %. Antagonistic bacterial strains of class *Firmicutes* were placed in separate cluster belonging to the genus *Bacillus*. In this study, two antagonistic endophytic bacterial strains were also identified showing low 16S rRNA gene sequence similarity (<98%). One strain of *Actinobacteria* i.e., *Streptomyces* sp. (EA64) and *one* strain belonging to *Firmicutes* i.e., *Bacillus* sp. (EA68) were identified with similarities of <98% to the closest type strains (Table 3). Four antagonistic *Actinobacteria* were selected for secondary metabolites identification depending on their antagonistic activity against oomycetes.

**Table 3:** Tentative novel taxa based on partial 16S rRNA gene sequence analyses from this study.

**Source**	**Similarity**	**Identity%**	**Family**	**Gram stain**
Rhizosphere (Soil)	*Bordetella avium* ATCC 35086 ^T^	98.9	*Alcaligenaceae*	Gram-negative
Rhizosphere (Soil)	*Nocardioides deserti* SC8A-24 ^T^	98.9	*Nocardioidaceae*	Gram-positive
Endophytic (Root)	*Streptomyces phaeopurpureus* NRRL B-2260 ^T^	98.8	*Streptomycetaceae*	Gram-positive
Endophytic (Root)	*Streptomyces diastatochromogenes* ATCC 12309^T^	97.3	*Streptomycetaceae*	Gram-positive
Endophytic (Root)	*Bacillus sonorensis* NBRC 101234 ^T^	95.7	*Bacillaceae*	Gram-positive
Endophytic (Leaf)	*Streptomyces europaeiscabiei* KACC 20186^T^	98.2	*Streptomycetaceae*	Gram-positive
Endophytic (Leaf)	*Bacillus halosaccharovorans* E33^T^	98.9	*Bacillaceae*	Gram-positive

### Enzymatic activities of antagonistic bacteria

Antagonistic bacteria were evaluated for their ability to produce fungal cell wall lytic enzymes. Protease, amylase, lipase, and cellulase activities of these bacteria were examined (Table 2). The number of bacteria exhibiting protease activity (*n* = 9; 41%) was high as compared to other enzymatic activities. Most of protease producing bacteria were endophytes (*n* = 6) and recovered from leaves and roots of halophyte. Amylase activity was observed for 7 (32%) antagonistic bacteria. Mostly strains of *Actinobacteria* showed amylase activity. Endophytic strains of *Actinobacteria* exhibited high production of amylase. Lipase activity was observed in 7 (32%) antagonistic bacteria. Strains of *Actinobacteria* and *Firmicutes* showed high production of protease. Cellulase activity was observed in only 1 (4.5%) bacterial strain. A strain of *N. dassonvillei* (EA52) exhibited cellulase activity belonged to *Actinobacteria*.

### Detection of PKS and NRPS genes

Bacteria possess NRPS/PKS gene clusters especially common in the phyla *Proteobacteria, Actinobacteria* and *Firmicutes*. Antagonistic bacterial isolates from this study showed that of 22 isolates, PCR amplification revealed that 13 strains (59%) were positive for at least one of the biosynthetic genes checked (Table 2). For the NRPS gene, 8 (36%) strains were positive. NRPS genes were detected in both classes of *Actinobacteria* and *Firmicutes*. The PKS-I gene was detected in only 4 (18%) antagonistic bacterial strains. While PKS-II gene was detected in 7 (32%) strains. All three genes PKS-I and PKS-II and NRPS were detected in strain *Bacillus* sp. (EA72). Three isolates belong to *Actinobacteria, Streptomyces enissocaesilis* sp. (EA52), *Streptomyces* sp. (EA63), and *Streptomyces* sp. (EA64) were positive for the presence of both NRPS and PKS-II.

### Identification of strain EA52, EA58 (rhizobacteria), and EA65, EA67 (endobacteria) metabolites by LC-MS

**Figure 2:**
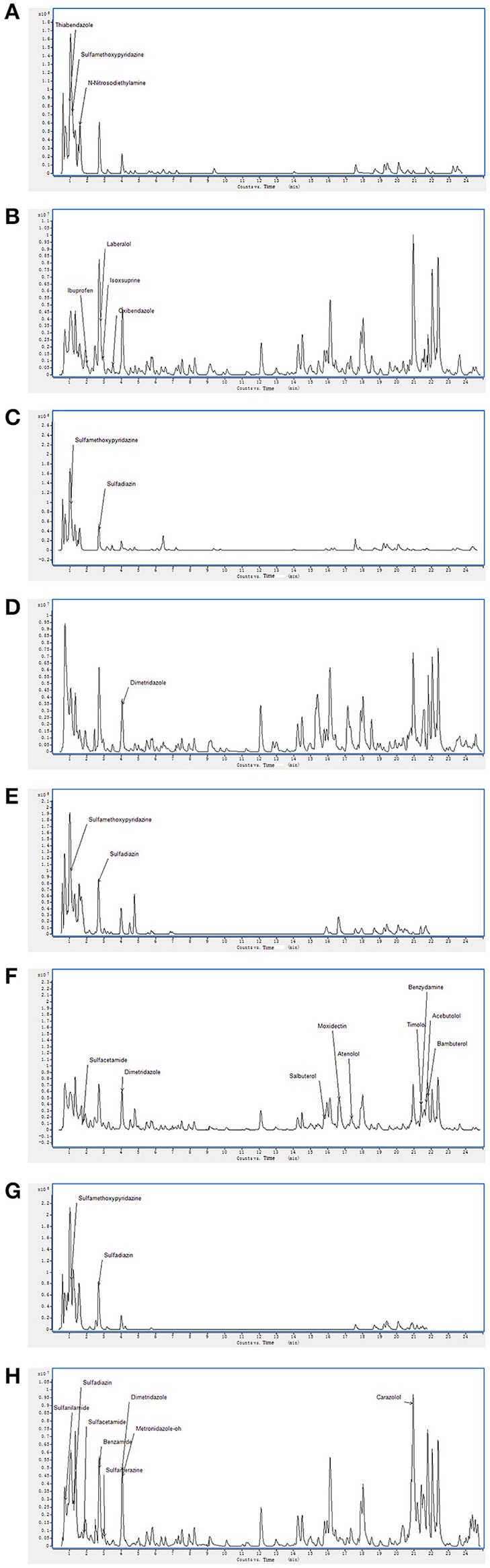
Spectra of LC/MS analysis showing detection of various bioactive metabolites. *Nocardiopsis* sp. (EA52) **(A)** negative mode LC/MS analysis **(B)** positive mode LC/MS analysis **(C)** negative mode LC/MS analysis of *Pseudonocardia* sp. (EA58) **(D)** positive mode LC/MS analysis **(E)** negative mode LC/MS analysis of *Streptomyces* sp. (EA65) **(F)** positive mode LC/MS analysis **(G)** negative mode LC/MS analysis of *Streptomyces* sp. (EA67) **(H)** positive mode LC/MS analysis.

On the basis of high inhibition activity against fungal pathogens we have selected four antagonistic bacterial strains from our study to identify secondary metabolites. These four antagonistic isolates belong to class *Actinobacteria* i.e., *Nocardiopsis* sp. (EA52) and *Pseudonocardia* sp. (EA58) were rhizobacteria while *Streptomyces* sp. (EA65) and *Streptomyces* sp. (EA67) are endophytes. Production of antifungal compounds was influenced by culturing in R2A as culture medium using optimum culture conditions. The maximum antifungal activity was observed after 48 h of growth at 28°C with pH 7.5. Metabolites in cultures extract of selected bacteria were analyzed using the LC-MS. LC-MS technique confirms presence of various bioactive compounds although not novel but already known for their bioactivity. Identification of metabolites was determined by LC-MS analysis and comparing results from NIST database. LC-MS analysis of culture extract of these selected strains identified different bioactive metabolites (Table 4). Strain of *Nocardiopsis* sp. (EA52) produced 7 identified peaks of different bioactive metabolites in both the positive- and negative-ion mode (Figures 2A,B). These compounds include Thiabendazole, Sulfamethoxypyridazine, N-Nitrosodiethylamine, Ibuprofen, Laberalol, Isoxsuprine, and Oxibendazole. For *Pseudonocardia carboxydivorans* (EA58), 2 bioactive compounds have been detected among hundreds of peaks known for other different metabolites. These three compounds include Sulfamethoxypyridazine and Dimetridazole (Figures 2C,D). Strain EA65 genetically closely associated as a *Streptomyces diastatochromogenes* showed presence of nine different bioactive compounds in the culture extract i.e., Sulfamethoxypyridazine, Dimetridazole, Salbutero, Moxidectin, Atenolol, Timolol, Benzydamine, Acebutolol, and Bambuterol (Figures 2E,F). These bioactive secondary metabolites are already known for their biological activities. LC-MS analysis of strain EA67, *Streptomyces europaeiscabiei* showed the presence of six bioactive compounds among several compounds detected. These bioactive compounds include, Sulfamethoxypyridazine, Benzamide, Sulfamerazine, Dimetridazole, Metronidazole-oh, and Carazolol (Figures 2G,H). These compounds are already known for antimicrobial, antiphytopathogenic and biocontrol properties.

**Table 4:** Secondary metabolites found in crude extract of selected four *Actinobacteria* strains.

**No**	**Name**	**Formula**	**RT**	**m/z**	**MASS**	**Score**	**Diff(DB, ppm)**	**Mode**	**Height**	**Area**
**STRAIN EA52**										
1	Thiabendazole	C10 H7 N3 S	0.962	201.037	201.0366	44.89	−2.4	Negative	36878	184023
2	Sulfamethoxypyridazine	C11 H12 N4 O3 S	1.125	279.0582	280.0655	75.22	−9.01	Negative	44633	255086
3	N-Nitrosodiethylamine	C4 H10 N2 O	1.579	215.0641	102.0784	46.98	8.48	Negative	23538	232204
4	Ibuprofen	C13 H18 O2	2.042	189.127	206.1303	92.03	2.07	Positive	62696	509785
5	Laberalol	C19 H24 N2 O3	2.8	329.1839	328.1766	81.9	6.46	Positive	54189	463656
6	Isoxsuprine	C18 H23 N O3	2.892	302.1733	301.1658	82.96	6.73	Positive	139154	2034111
7	Oxibendazole	C12 H15 N3 O3	3.533	250.1187	249.1114	97.67	−0.32	Positive	126155	961667
**STRAIN EA58**										
8	Sulfamethoxypyridazine	C11 H12 N4 O3 S	1.117	279.0575	280.0647	67.12	−6.21	Negative	15321	77965
9	Sulfadiazin	C10 H10 N4 O2 S	2.683	249.0475	250.055	72.64	−10.1	Negative	29954	210781
10	Dimetridazole	C5 H7 N3 O2	4.045	159.0876	141.0527	70.14	8.14	Positive	111793	948315
**STRAIN EA65**										
11	Sulfamethoxypyridazine	C11 H12 N4 O3 S	1.126	279.0574	280.0649	80.47	−6.78	Negative	47164	293920
12	Sulfadiazin	C10 H10 N4 O2 S	2.686	249.0474	250.0548	74.78	−9.52	Negative	41010	344049
13	Sulfacetamide	C8 H10 N2 O3 S	1.793	215.0474	214.0401	72.66	5.06	Positive	68031	563882
14	Dimetridazole	C5 H7 N3 O2	4.039	159.0881	141.0535	82.65	2.25	Positive	211556	1889105
15	Salbuterol	C13 H21 N O3	15.786	239.1764	239.1532	47.28	−4.28	Positive	1181230	8578877
16	Moxidectin	C37 H53 N O8	16.671	662.3704	639.3739	49.37	4.97	Positive	95729	1314871
17	Atenolol	C14 H22 N2 O3	17.318	267.1729	266.1657	77.62	−10.11	Positive	907768	7927165
18	Timolol	C13 H24 N4 O3 S	21.415	317.1641	316.1571	73.26	−0.54	Positive	67063	538273
19	Benzydamine	C19 H23 N3 O	21.419	310.1884	309.1797	21.59	14.35	Positive	89361	929352
20	Acebutolol	C18 H28 N2 O4	21.711	336.2052	336.2045	59.81	1.09	Positive	88941	887397
21	Bambuterol	C18 H29 N3 O5	21.714	367.2376	367.2098	51.49	2.53	Positive	58314	570222
**STRAIN EA67**										
22	Sulfamethoxypyridazine	C11 H12 N4 O3 S	1.122	279.0576	280.0651	80.42	−7.33	Negative	30594	156782
23	Sulfadiazin	C10 H10 N4 O2 S	2.682	249.0471	250.0545	78.19	−8.26	Negative	44098	367641
24	Sulfanilamide	C6 H8 N2 O2 S	0.748	173.0376	172.0307	91.12	−0.47	Positive	95732	1075748
25	Sulfadiazin	C10 H10 N4 O2 S	1.223	251.058	250.0508	65.17	6.67	Positive	99002	476334
26	Sulfacetamide	C8 H10 N2 O3 S	1.8	215.0475	214.0404	90.98	3.98	Positive	142535	1446212
27	Benzamide	C7 H7 N O	2.72	103.0426	121.0525	37.43	1.82	Positive	641812	5370210
28	Sulfamerazine	C11 H12 N4 O2 S	2.991	265.0734	264.0662	65	7.1	Positive	59659	389438
29	Dimetridazole	C5 H7 N3 O2	4.059	159.0879	141.0531	75.55	5.01	Positive	149866	1286924
30	Metronidazole–oh	C6 H9 N3 O4	4.06	188.0669	187.0597	95.68	−1.89	Positive	1776794	17824964
31	Carazolol	C18 H22 N2 O2	20.924	303.1474	298.1688	42.24	−2.22	Positive	81193	668836

## Discussion

There is a need for the discovery of new drugs due to the emergence of resistant bacteria to different antibiotics and cancer cells to anticancer drugs. Currently, marine habitats have been identified as a source of discovering many important novel therapeutic agents (Malve, 2016). *S.imbricata*, is a halophyte containing various chemical compounds of medicinal use and it is widely spread in the sea shore areas in Saudi Arabia. Generally halophytes with traditional health benefits in folk medicine are a source of attraction for modern comprehensive microbial and chemical investigations. In our previous study (Bibi et al., 2017) we have isolated two rhizobacteria from *Salsola* exhibiting cellulase and amylase activity on culturing media. One strain EA151 with cellulase activity belonged to *Firmicutes* while other strain EA152 exhibited amylase activity closely related to type strain of *Actinobacteria*.

In this study, rhizopheric and endophytic bacterial populations with potent antagonistic activity against phytopathogens were studied. One hundred and sixty eight rhizopheric and endophytic bacteria were isolated and only 22 were found to be potent antagonists of oomycetes. Different cultivation media were used to culture bacteria to isolate novel isolates with unique bioactivities. A study has reported that high nutrient medium favors growth of diverse groups of bacteria, while low nutrient media increase number of bacteria (Vishnivetskaya et al., 2000). To our knowledge, this work is the first study on the isolation, screening, characterization and metabolites identification of both rhizospheric and endophytic bacteria from *S. imbricata*. In this study, culture-dependent approach was used to study the diversity of antagonistic bacteria from halophyte against oomycetes plant pathogens. Screening of 168 rhizospheric and endophytic bacteria and their 16S rRNA gene sequencing resulted in 22 different bacteria belong to four major classes. Bioactive compounds production by rhizo and endophytic bacteria is one of the phenomenon used by bacteria to defend the host against different pathogens.

Antagonistic bacteria belong to four major classes, *Firmicutes*, γ*-Proteobacteria*, and β*-Proteobacteria*. Our results show that *Actinobacteria* is recognized as a dominant phylum among these antagonitistic bacteria isolated from halophyte (Table 1). Actinobacteria are distributed in various ecological habitats especially in terrestrial and aquatic ecosystems where they play a potential role in decomposition of plants and animals remains. They are biotechnologically important due to their ability to produce secondary metabolites (Berdy, 2005). Therefore, we have selected four different strains of *Actinobacteria* for further study of bioactive metabolites produced by these antagonistic rhizopheric and endophytic bacteria. Results of 16S rRNA sequence analyses showed a diversity of different genera of *Actinobacteria* and the genus *Streptomyces* remain which are dominant in our study. Results of this study are also compatible with previous studies where many novel *Actinobacteria* have been recovered from different unexplored marine sources (Fiedler et al., 2005; Bull and Stach, 2007). Halophytes growing near the sea shore in tropical and subtropical areas have become valuable sources for the isolation of microbial flora especially in the group *Actinobacteria* because of the chemodiversity of environmental factors prevailing there. The presence of major numbers of *Streptomyces* strains amongst antagonists from the halophyte in this study are in accordance with the results reported previously (Eccleston et al., 2008; Ravikumar et al., 2012). In *Actinibacteria*, the most active bacterial strains in addition to *Streptomyces* are, *Nocardiopsis, Pseudonocardia*, and *Arthrobacter* which have been reported for their inhibitory activities and production of antimicrobial metabolites (Anandan et al., 2016).

The metabolic diversity of *Streptomycetes* is remarkable and novel strains with new bioactive compounds including antibiotics have been isolated from extreme habitats (Fiedler et al., 2005). Until now, several studies have reported diverse groups of antimicrobial compounds by bacteria isolated from halophytic endophytes, such as steroids, peptides, alkaloids, terpenoids, quinines, flavonoids, and phenols (Newman and Cragg, 2007). Our results showed that most of the endophytes in this study belonging to *Streptomyces* and were recovered from root samples. This finding is supported by previous results where maximum number of endophytes were recovered from roots specimens (Taechowisan et al., 2003; Passari et al., 2015) This may be due to the reason that the rhizosphere has a high concentration of nutrients and various bacteria enter different root tissues of the host plant through the rhizosphere and become endophytic in their mode of life (Rosenblueth and Martinez-Romero, 2006).

The second dominant class of bacteria in this study was *Firmicutes*. The group of bacteria belonging to the class *Firmicutes* are commonly found as they are easily culturable. Also, from marine sources, species of *Bacillus* are dominant for the production of antibacterial, antifungal antibiotics, surfactants, and lytic enzymes (Mondol et al., 2013). Endophytic bacterial strains of *Bacillus* from halophytes are already reported to show inhibition against bacterial and fungal plant pathogens (Menpara and Chanda, 2013). In this study, only two antagonistic rhizopheric and endophytic bacteria were related to the class *Proteobacteria* belonging to two different classes γ*-Proteobacteria* and β*-Proteobacteria*. Members of class γ*-Protobacteria* from marine source produce the highest number of secondary metabolites (Long and Azam, 2001).

Hydrolytic enzymes from marine bacteria are produced and used by them in catalyzing different biochemical processes in the marine environment (Thatoi et al., 2013). In addition to production of useful secondary metabolites and antibiotics Actinobacteria from marine sources are also known for the production of lytic enzymes of industrial use (Anandan et al., 2016). In this study, mostly strains of *Actinobacteria* and *Firmicutes* were active candidates for production of different enzymes. Protease, lipase and amylase activity was mostly observed (Table 2). Only one strain EA59 exhibited protease, amylase and lipase activity. This strain is endophytic and belongs to *Actinobacteria*. It seems as if the productions of these hydrolytic enzymes is necessary for antagonism as well as for intracellular colonization of bacteria in the host plant. Only four strain of rhizobacteria belong to *Actinobacteria* were able to produce at least one type of enzyme tested in this study. While most of the endophytic bacteria were able to show enzymatic activities belong to *Actinobacteria*. Marine *Actinobacteria* have the potential to produce extracellular enzymes of industrials use. These extracellular enzymes are usually used by endophytic bacteria for their antagonism against different pathogens invading host plants (Zhao et al., 2016).

Polyketide synthetase (PKS) and nonribosomal polyketide synthetase (NRPS) genes are considered as hallmarks for the production of secondary metabolites. These biosynthetic genes of bacterial secondary metabolites are involved in the biosynthesis of many bioactive products. The presence of the biosynthetic genes PKS and NRPS in rhizospheric and endophytic bacteria associated with this halophyte suggests that these beneficial bacteria are dominant producers of bioactive natural products. Amplification of these three biosynthetic gene clusters domains is an indirect approach to test the knowledge that bioactive products are being produced by the organism. Of 22 isolates screened for detection of PKS-I, PKS-II, and NRPS gene, only 13 strains were positive for at least one of the biosynthetic genes tested. Several species of *Streptomyces* possess two types of PKSs. One is responsible for antibiotic synthesis, while the other is involved in spore pigments production in various *Streptomyces* species (Bergh and Uhlén, 1992). In this study, PKS-II and NRPS were detected in most of the *Streptomyces*. Among them, strains EA52, EA61, EA63, and EA64 were positive for the presence of NRPS and PKS-II, and also exhibited antifungal activity against tested pathogens (Table 2). While strains EA53, EA56, EA59, EA60, EA62, EA68-EA71 were negative for the presence of PKS-I, PKS-II, and NRPS genes and positive for antifungal activity. Therefore, there is no correlation between presence of PKS and NRPS gene and antimicrobial properties (Qin et al., 2009). It is evident that NRPS PKS-I and PKS-II gene screening using PCR in different bacterial strain help to find out potential strain of bacteria producing important secondary metabolites.

Strains of *Streptomycetes* are active producers of antimicrobial compounds and more than 70% of antibiotics in the world are produced by them. Endophytic *Streptomycetes* occupy a biologically important niche in tissues of the host plant, taking nutrition from host and in turn providing protection to the plant. These endophytes produce important metabolites that are not toxic to their host therefore, can be considered as important bioactive metabolites in drug discovery (Castillo et al., 2007). Selection of *Actinobacteria* for identification of active secondary metabolites resulted in a number of known synthetic bioactive compounds not of microbial origin (Table 4). Based upon antifungal activity and low 16S rRNA gene similarity four out of twenty-two rhizopheric and endophytic strains were ultimately selected for LC-MS analyses. Production of antifungal compounds was influenced by culturing the organism in R2A as a culture medium using optimum culture conditions. The maximum antifungal activity was observed after 48 h of growth with pH 7.5 at 28°C. Metabolites in cultures extracts of selected bacteria were analyzed using LC-MS. Two rhizopheric strains namely EA52 closely related to *Nocardiopsis* sp. and EA58 showed similarity with *Pseudonocardia* sp. in producing diverse metabolites including Thiabendazole, Sulfamethoxypyridazine, N-Nitrosodiethylamine, Ibuprofen, Laberalol, Isoxsuprine, Oxibendazole, and Dimetridazole. Strain EA52 showed close similarity to *Nocardiopsis sp*. This marine non-Streptomycete genus of *Actinobacteria*, contained a variety of bioactive compounds and showed a wide range of activities including cytotoxicity, antibacterial, antifungal, and anti-angiogenesis. *Nocardiopsis sp*. contained α-pyrone (nocapyrone S1) and (4-aminophenyl) acetic acid, N-(2- hydroxyphenyl)-acetamide, cyclo-(L-Pro-L-Val), cyclo-(L-Pro-L-Leu), and cyclo-(L-Pro-L-Ile) when evaluated for secondary metabolite production (Zou et al., 2017) but different secondary metabolites have been detected in closely related strain EA58 in the present study (Figures 2A,B). Antimicrobial activity of strains of *Pseudonocardia* is already evident from previous studies but no such antifungal activity and metabolites detected in our study were produced from the closely related strain EA58 (Figures 2C,D; Tanvir et al., 2016).

Two endophytic *Streptomycetes* EA65 and EA67 were analyzed by LC-MS and they exhibited spectra for 15 bioactive compounds including antibacterial, antifungal, antiprotozoal and anthelmintic compounds. Strain EA65 showed close similarity at the 97% level to a rhizobacterium *S. diastatochromogenes*, a promising source of antifungal metabolites and novel natural antibiotics and used as biocontrol agents (Shentu et al., 2016). *S. diastatochromogenes* reportedly produces polyketomycin, momofulvenoneA, azdimycin, toyocamycin, concanamycin, and oligomycins. Strain EA65 exhibited the presence of bioactive compounds not reported in *S. diastatochromogenes* including Sulfamethoxypyridazine, Dimetridazole, Salbutero, Moxidectin, Atenolol, Timolol, Benzydamine, Acebutolol, and Bambuterol (Figures 2E,F). Another endophytic strain EA67 related to *S. europaeiscabiei* showed the presence of bioactive compounds such as Sulfamethoxypyridazine, Benzamide, Sulfamerazine, Dimetridazole, Metronidazole-oh, and Carazolol (Figures 2G,H). These compounds are already known for antimicrobial, antiphytopathogenic and biocontrol properties. Sulfamethoxypyridazine, Nitroimidazole including Dimetridazole and Metronidazole-oh and Moxidectin are major antimicrobial compounds detected in all four strains in this study. Sulfamethoxypyridazine was detected in all four strains in our study. These antibacterial sulfonamides are synthetic antimicrobial agents used as antibiotics in different inflammatory diseases (Vicente and Pérez-Trallero, 2010). This is first study showing the isolation and screening of antagonistic bacteria from *S. imbricata* and the identification of active metabolites from crude extracts. Compounds produced by these strains although not new, have been not reported in literature from other organisms and most of them are synthetic so this is the first reported of these *Actinobacteria* as a natural source for production of these antimicrobial compounds.

In conclusions, present study of antagonistic bacteria from *S. imbricata* exhibited wide range antifungal and enzymatic activities indicating their industrial, biotechnological and agricultural potential. Further analyses of bioactive metabolites from selected *Actinobacteria* exhibited production of known antibiotics and bioactive compounds of synthetic nature not reported from bacteria before. These results suggest that halophytes are potential source of bioactive metabolites via their associated bacterial microflora. This study points to an enormous potential of discovering novel bioactive compounds using the methodology described. Currently we are working on some selected novel strains for deep bioactive analysis and genome sequencing that will be presented in our future work.

## Author contributions

FB and GS: designed the study and write manuscript; MN and EA: identified the plant samples and help in collection of sample and experimental work; MY and AK collect and analyzed the data. All authors read and approved the final manuscript.

### Conflict of interest statement

The authors declare that the research was conducted in the absence of any commercial or financial relationships that could be construed as a potential conflict of interest.
